# The Emerging Genetic Landscape of Hirschsprung Disease and Its Potential Clinical Applications

**DOI:** 10.3389/fped.2021.638093

**Published:** 2021-08-05

**Authors:** Anwarul Karim, Clara Sze-Man Tang, Paul Kwong-Hang Tam

**Affiliations:** ^1^Department of Surgery, Li Ka Shing Faculty of Medicine, The University of Hong Kong, Hong Kong, China; ^2^Li Dak-Sum Research Center, The University of Hong Kong—Karolinska Institute Collaboration in Regenerative Medicine, Hong Kong, China

**Keywords:** Hirschsprung disease, aganglionosis, genetics, genetic architecture, rare variants, GWAS, next-generating sequencing, common variants

## Abstract

Hirschsprung disease (HSCR) is the leading cause of neonatal functional intestinal obstruction. It is a rare congenital disease with an incidence of one in 3,500–5,000 live births. HSCR is characterized by the absence of enteric ganglia in the distal colon, plausibly due to genetic defects perturbing the normal migration, proliferation, differentiation, and/or survival of the enteric neural crest cells as well as impaired interaction with the enteric progenitor cell niche. Early linkage analyses in Mendelian and syndromic forms of HSCR uncovered variants with large effects in major HSCR genes including *RET, EDNRB*, and their interacting partners in the same biological pathways. With the advances in genome-wide genotyping and next-generation sequencing technologies, there has been a remarkable progress in understanding of the genetic basis of HSCR in the past few years, with common and rare variants with small to moderate effects being uncovered. The discovery of new HSCR genes such as neuregulin and *BACE2* as well as the deeper understanding of the roles and mechanisms of known HSCR genes provided solid evidence that many HSCR cases are in the form of complex polygenic/oligogenic disorder where rare variants act in the sensitized background of HSCR-associated common variants. This review summarizes the roadmap of genetic discoveries of HSCR from the earlier family-based linkage analyses to the recent population-based genome-wide analyses coupled with functional genomics, and how these discoveries facilitated our understanding of the genetic architecture of this complex disease and provide the foundation of clinical translation for precision and stratified medicine.

## Introduction

Hirschsprung disease (HSCR), also known as colonic aganglionosis, is the leading cause of neonatal functional intestinal obstruction. It is a rare congenital developmental defect of the enteric nervous system (ENS) with a global incidence of 1 in 3,500–5,000 live births. The incidence of the disease varies widely among ethnic groups and is the highest among Asians (2.8/10,000 live births) (1, 2). The disease is characterized by the absence of enteric ganglia in the distal colon due to the failure of enteric neural crest cells (ENCCs) to fully colonize the hindgut during embryonic development. The incomplete innervation may result from any or a combination of genetic defects and environmental factors affecting migration, proliferation, differentiation, and survival of the ENCCs as well as impaired extracellular milieu or impaired interaction between the migrating neural crest-derived precursor cells and the mesenchymal microenvironment through which they migrate (3, 4).

HSCR is traditionally classified by the extent of aganglionosis. In short-segment HSCR (S-HSCR; 80% of cases), aganglionosis does not extend beyond the sigmoid region whereas in long-segment HSCR (L-HSCR; 20% of cases), it extends proximal to the sigmoid colon. Among the L-HSCR, if the aganglionosis extends to at least ileocecal valve, it is termed total colonic aganglionosis (TCA), which represents approximately 5% of all HSCR cases. The majority of HSCR cases (~70%; isolated HSCR) occur as isolated anomalies. Other cases are syndromic (syndromic HSCR) and often encountered with chromosomal aberrations and/or a range of other congenital malformations (1, 2, 5–8). The inheritance of HSCR is considered to be complex. It most commonly presents as sporadic forms (80–90%), which are more often S-HSCR and follow a multifactorial inheritance pattern. The remaining 10–20% of cases are familial and tend to be of L-HSCR/TCA with autosomal dominant inheritance (2, 9). HSCR also exhibits significant sex bias with a marked male preponderance. Especially for S-HSCR, the disease prevalence is four times higher in males than in females. Up till now, the only definitive treatment available for HSCR is surgery.

Over the past decades, through human genetic studies—beginning with the classical positional cloning, linkage analyses, and candidate gene screening—through genome-wide association studies (GWAS)—to the recent next-generation sequencing (NGS) studies, coupled with validation by functional genomic studies, many genetic variants and genes have been linked to HSCR. Majority of these genes belong to several key biological pathways that often crosstalk and have been shown to orchestrate the dynamic process of ENS development and HSCR pathogenesis, though some initially unsuspected candidate genes have also been uncovered by the bias-free approaches. These studies have provided solid evidence that most HSCR cases have complex (oligogenic/polygenic) genetic basis wherein multiple HSCR-associated common variants contribute to the genetic predisposition and modify the penetrance of rare damaging variants in disease-relevant genes and hence disease manifestation.

In this review, we explore the paradigm shift in genetic discoveries of HSCR from family-based, candidate gene studies to population-based, genome-wide analyses with the advent of genotyping and sequencing technologies and how these discoveries furthered our understanding of the genetic architecture of this complex disorder. This review primarily focuses on the emerging genetic landscape of rare coding variants, common regulatory variants, and the interplay between them as well as their differential contribution to HSCR and the disease subtypes, and how the new knowledge may pave the way for genome data to be incorporated as part of “routine” in the precision-medicine era.

## Pre-GWAS ERA: Identification of Core HSCR Pathways Through Linkage Mapping and Candidate Gene Studies on Syndromic and Familial HSCR Cases

Genetic analyses to uncover the genes underlying HSCR began in late 1980s/early 1990s. Table 1 lists the main genes known to be involved in either syndromic or isolated HSCR. In the early days, analyses were primarily focused on the more Mendelian forms of syndromic and familial HSCR. These studies built upon the fundamental idea that disease causal variant and nearby genetic markers tend to be transmitted together due to linkage disequilibrium (LD). Such approach of positional cloning and linkage analysis have been applied to multiplex families where highly informative genetic markers are used to map the disease-associated loci of large effect. Once a locus is linked, a search for rare damaging mutations (i.e., variants with minor allele frequency <1% in general population) in candidate genes within the locus is ensued. This strategy remained very popular especially before the GWAS era. In fact, the two major HSCR genes—*RET* (in 10q11.2) and *EDNRB* (in 13q22.3)—representing the two core HSCR pathways as well as transcription factors underlying syndromic form of HSCR were initially discovered with this approach in the early 1990s (Table 1).

**Table 1:** Genes reported with rare damaging protein-altering variants in patients with HSCR.

**Gene**	**Phenotype**	**Frequency^a^**	**References**
*RET*	Isolated HSCR	50% familial 15–20% sporadic	(10–13)
	MEN2A with HSCR		
	MEN2B with HSCR		
	FMTC with HSCR		
*GDNF*	Isolated HSCR	Rare	(14–16)
*NRTN*	Isolated HSCR	Very rare	(17)
*ARTN*	Isolated HSCR	Very rare	(17)
*PSPN*	Isolated HSCR	Very rare	(17)
*GFRA1*	Isolated HSCR	Rare	(16, 18)
*EDNRB*	Isolated HSCR	Rare	(16, 19)
	Shah-Waardenburg syndrome		
*EDN3*	Isolated HSCR	Very rare	(18)
	Shah-Waardenburg syndrome		
*ECE1*	HSCR with cardiac, craniofacial, and autonomic defects	Very rare	(20)
*SOX10*	Isolated HSCR	Very rare	(21)
	Shah-Waardenburg syndrome		
*PHOX2B*	Haddad syndrome (Congenital Central Hypoventilation Syndrome with HSCR)	Very rare	(22)
	Neuroblastoma with HSCR	Very rare	
	HSCR with dysmorphic facial features	Very rare	
*ZEB2*	Mowat–Wilson syndrome	Very rare	(23)
*KIAA1279(KIFBP)*	Goldberg–Shprintzen syndrome	Very rare	(24–26)
*NRG1*	Isolated HSCR	Rare	(27)
*ERBB2*	Isolated HSCR	Rare	(16)
*SEMA3C/D*	Isolated HSCR	Rare	(18)
*IHH*	Isolated HSCR	Very rare	(28)
*GLI1*	Isolated HSCR	Very rare	(29)
*GLI2*	Isolated HSCR	Very rare	(29)
*GLI3*	Isolated HSCR	Very rare	(28, 29)
*L1CAM*	X-linked hydrocephalus	Very rare	(30)
*ITGB4*	Isolated HSCR	Rare	(16)
*PTK2*	Isolated HSCR	Rare	(16)
*DENND3*	Isolated HSCR	Very rare	(31)
*NCLN*	Isolated HSCR	Very rare	(31)
*NUP98*	Isolated HSCR	Very rare	(31)
*TBATA*	Isolated HSCR	Very rare	(31)
*VCL*	Isolated HSCR	Very rare	(32)
*BACE2*	Isolated HSCR	Rare	(16)
*ACSS2*	Isolated HSCR	Rare	(18)
*ENO3*	Isolated HSCR	Rare	(18)
*SH3PXD2A*	Isolated HSCR	Rare	(18)
*UBR4*	Isolated HSCR	Rare	(18)

*Table is updated and adapted from (2) and (33)*.

a *Rare: Variants detected in 1–7% of patients with HSCR screened and reported*.

*Very rare: Variants detected in <1% of the patients with HSCR screened and reported*.

### RET Signaling

*RET* (Rearranged during Transfection) proto-oncogene encodes a transmembrane tyrosine-protein kinase receptor. It is activated by glial cell line-derived neurotrophic factor (GDNF) family ligands, such as GDNF, NRTN (neurturin), ARTN (artemin), PSPN (persephin), and co-receptors named GDNF-family receptor-α (GFRα1-4) (34). *RET* plays a pivotal role in both isolated and syndromic HSCR. The *RET* locus was first identified as a susceptibility locus for HSCR through linkage analyses in multiplex HSCR families (35, 36) facilitated by the finding of deletion of the proximal long arm of chromosome 10 in patients with isolated HSCR (37, 38) and the co-occurrence of HSCR and multiple endocrine neoplasia type 2 (MEN2) (39, 40). This was followed by the identification of numerous *RET* mutations, including missense, splicing variants, and short insertions and deletions (indels), across the whole spectrum of patients with HSCR occurring both as *de novo* and inherited events (10–13, 41–52). Damaging coding mutations in *RET* are identified in approximately 50% of familial and 15–20% of sporadic HSCR cases (1, 10–13), and it gradually becomes evident that rare coding variants in *RET* appear to play a less prominent role in sporadic and S-HSCR compared to the familial and L-HSCR (51, 53). Furthermore, somatic *RET* mutations have also been reported occasionally—occurring either as transmission of mosaic germline mutation from unaffected parent to affected patient or as somatic mutation in patients (54–57).

Subsequent to the discovery of *RET* in HSCR pathogenesis, mutation screening was performed on interacting partners and other key members of RET signaling pathway with reference to known biological processes and animal models. Damaging rare variants in all four ligands of RET have been detected in patients with HSCR, yet *RET* still contributes to the vast majority of rare variants reported in genes in this core HSCR pathway (13–15, 17, 28, 51, 58–62). Mutations in these GDNF family ligands typically co-occurred with variants in other major HSCR genes, particularly in *RET*, except in a few instances (59, 61). As for those exceptions, the patients may also carry variants in other HSCR genes not yet discovered by that time. This is exemplified by a recent whole exome sequencing (WES) study by Sribudiani et al. (28) where the authors showed that a *de novo* in-frame deletion in *GDNF* was, together with rare inherited variants in *IHH* (Hedgehog signaling pathway, described later) and its mediator, *GLI3*, responsible for HSCR in a branch of a multigenerational HSCR family. Similarly for the co-receptors, no confirmed high-penetrant pathogenic variant has yet been described in patients except a marginal increase in burden of rare damaging protein-altering variants in *GFR*α*1* that has been detected in a recent whole-genome sequencing (WGS) study on 443 East Asian S-HSCR cases compared to 493 controls (16, 18, 63). Altogether, these findings suggest that, in the majority of cases where variants are found in GDNF family ligands and co-receptors, they alone are not sufficient to cause HSCR and rather act as modifiers together with other risk variants (rare damaging or common regulatory risk variants) in a digenic/oligogenic pattern.

### EDNRB Signaling

*EDNRB* (Endothelin receptor type B) gene encodes a non-specific G protein-coupled receptor for endothelins (EDN1, EDN2, and EDN3) (2). After synthesis, endothelins are converted into a shorter active form by endothelin converting enzyme (ECE-1) (2). Similar to *RET*, the contribution of *EDNRB* to the risk of HSCR was discovered by family-based studies. A HSCR susceptibility locus was first mapped to 13q22 by identity-by-descent and linkage analysis in a large, inbred, Mennonite kindred that manifested high incidence of HSCR and pigmentary disorders, the Waardenburg syndrome type 4 (WS4) (64). A follow-up study identified p.Trp276Cys mutation in *EDNRB* that showed incomplete penetrance and dosage effect in addition to being absent in some patients (65). This was followed by several reports of identification of *EDNRB* mutations in patients with HSCR and WS4 syndrome (11, 66–70). However, rare damaging variants of *EDNRB* account for only 3–7% of isolated HSCR cases (2).

Subsequent search for rare variants in genes in EDNRB pathway reported pathogenic mutations in *EDN3* and *ECE1* in patients with HSCR. In general, homozygous mutations of *EDNRB* and *EDN3* are more commonly associated with WS4 and heterozygous mutations are more commonly associated with isolated HSCR (11, 66–73). Thus far, only one heterozygous *ECE1* variant has been reported in a syndromic patient with HSCR (20).

Although rare damaging variants in EDNRB pathway genes are encountered in only a small fraction of patients with HSCR (~5%), they generally exhibit higher penetrance and confer higher risk than variants in RET signaling pathway genes. For example, in a WES study by Tilghman et al. on European HSCR cases and controls (18), pathogenic variants in EDNRB pathway genes (seven in *EDNRB* and one in *EDN3*) were exclusively present in HSCR cases. In addition, pathway-based odds ratio (OR) for EDNRB was considerably higher [OR = 69.03; 95% confidence interval (CI): 8.68–547.92] than that of the RET signaling pathway (OR = 16.03; 95% CI: 5.21–49.28). Consistently, *EDNRB* variants showed an overall higher risk than *RET*, for S-HSCR cases in East Asian WGS analysis (16).

### Transcription Factors Critical for ENS Development

*SOX10* encodes a transcription factor that is a key regulator of ENS development (74) and is implicated in Waardenburg syndrome with varying spectrum of features with or without HSCR (75–77). Although *SOX10* variants were initially thought to cause HSCR as a part of WS4 (2), recent studies identified several isolated HSCR cases with *SOX10* mutations—both in its coding region (21, 78) and in its enhancers (78–80).

*PHOX2B* also encodes a transcription factor required for normal development of the ENS (81). Genetic defects in *PHOX2B* have been described primarily with congenital central hypoventilation syndrome (CCHS) (82), which occur with HSCR in 15–20% of cases (83) and in some reports with neuroblastoma (84, 85). These strongly suggest a pathogenic role of *PHOX2B* in HSCR. In terms of rare coding variants, a *de novo* in-frame deletion in *PHOX2B* in a female patient with L-HSCR and other anomalies but without clinical manifestations of CCHS or neuroblastoma has been described (22). However, unlike *SOX10, PHOX2B* pathogenic variants in isolated HSCR are yet to be found. In addition to rare variants, common *PHOX2B* polymorphisms were shown to be moderately associated with HSCR in a Chinese population (86, 87) and interaction between *RET* and *PHOX2B* polymorphisms was demonstrated to significantly increase HSCR risk (88).

*ZEB2*, previously known as *SIP1* or *ZFHX1B*, encodes a transcription factor that is crucial to direct the formation, migration, and specification of neural crest cells (89). Variants in this gene have been described with many patients with Mowat–Wilson syndrome, which includes HSCR in its phenotypic spectrum in approximately half of the cases (23). However, *ZEB2* pathogenic variants have not been described in isolated HSCR yet. No enrichment for rare variants in *ZEB2* was found in S-HSCR cases compared to controls, indicating that it is likely to be a very rare contributor to isolated HSCR (16).

### Genes Related to Cytoskeleton

Homozygosity mapping followed by sequence analysis in a consanguineous family with multiple members having Goldberg–Shprintzen syndrome (GOSHS) identified homozygous truncating mutations in *KIAA1279 (KIFBP)* (24). HSCR has been reported in 64% of patients with GOSHS (25). Thus far, 16 different variants in this gene have been described in patients with GOSHS (25). *KIAA1279* encodes for KIF-binding protein (KIFBP), which interacts with microtubule and actin filaments and plays a role in neurite outgrowth, neuronal development, and differentiation (90, 91). Mice null for this protein show delayed gut colonization by the neural crest-derived cells (92). KIFBP was shown to interact with several kinesins and SCG10 (a microtubule destabilizing protein), implicating the potential role of cytoskeleton/microtubule-related defects in HSCR pathogenesis (90). Related to this, it should also be noted that a slightly lower intensity of immunoreactivity of Microtubule-Associated Protein 5 (MAP5) was noted in the intestinal tissue of the aganglionic segment of patients with HSCR compared to the normoganglionic segment of the same patients or intestinal tissue of control individuals (93). However, aside from the *KIAA1279* variants in patients with GOSHS, no variant has been reported in *SCG10* (94) or other related genes yet in patients with HSCR.

## The ERA of GWAS: Large Contribution of Common Variants Implicating New Disease Pathways and Emerging Genetic Landscape

Altogether, the rare damaging variants identified in RET and EDNRB pathways explain only a small fraction (<30%) of sporadic HSCR cases. The inability to detect rare broadly Mendelian variants in considerable proportions of patients, along with the variable expressivity and disease severity among carriers, implied that more complex genetic etiologies are likely to underlie HSCR. Other genetic factors or epistatic regulation of *RET*, either by variants within the locus or with other unlinked genes, must exist to explain the missing heritability.

Contribution of common variants to HSCR was first indicated by (i) the variable penetrance of a damaging *RET* missense mutation (*RET* c.1859G>C;p.Cys620Ser) under different haplotype backgrounds of common variants in a multiplex family (95) and (ii) subsequent reports of overrepresentation of certain *RET* haplotypes in patients with HSCR across populations (96–98). Later, a study by Emison et al. pinpointed a common functional *RET* intron 1 enhancer variant (RET+3; rs2435357 T/C) that largely increases risk of HSCR (OR~5). The risk allele (T) of RET+3, present in ~60% of European and ~80% of Asian cases, significantly reduces *RET* expression and confers higher risk for HSCR than rare alleles collectively (99). It is interesting to note that the risk allele frequency is substantially higher in the general population in East Asians than Europeans (47 vs. 23%), which could explain the higher incidence of HSCR in Asia (53). In contrast to rare damaging variants, contribution of this common enhancer variant is considerably higher in the major subtype of sporadic and isolated S-HSCR than the severe forms (53). Furthermore, another *RET* intron 1 risk variant (rs2506004 A/C), which is in near complete LD with rs2435357 (r^2^ = 0.99 in Asians and 1 in Europeans) and separated by only 217 base pairs from it, was also identified (100, 101). The risk allele (A) alters *RET* expression by interfering with the binding of transcription factors NXF, ARNT2, and SIM2 to this locus exemplifying that more than one variant may be relevant to disease pathogenesis in the same disease-associated haplotype (101). Altogether, these findings implied that common variants can predispose to HSCR in a low penetrance manner by modifying the phenotypic expression, which opened up a new area of genetic research on HSCR, including family-based and population-based association studies by detecting transmission disequilibrium of common single-nucleotide polymorphisms (SNP) from parents to proband and comparing frequencies of SNPs in cases vs. controls, respectively (53, 102–110). However, earlier association studies still relied on prior biological knowledge to detect association of variants in candidate region(s) and, with this candidate gene approach, no confident association beyond the *RET* locus could be detected or replicated.

The advances of high-throughput SNP array, assaying hundreds of thousands of SNPs simultaneously, permitted the transition to a hypothesis-free approach for a powerful detection of association in genome-wide scale, termed GWAS. Thus far, five array-based GWAS have been published on HSCR, which unraveled two novel, highly replicable GWAS significant loci—*NRG1* and *SEMA3C/D—*in addition to *RET* (shown in Table 2) (102–108, 110). Transethnic meta-analysis of GWAS discovered universal as well as ancestry-specific risk alleles, highlighting the heterogeneity of the genetic architecture of this disease (111). Other common SNPs that were found with moderate associations in GWAS includes SNPs in or nearby *PHOX2B, JAG1*, and *VRK2*. Epistatic interaction within and between *RET* and other loci were discovered from these GWASs as well. Novel insights and pathways previously not linked to the genetic etiology of HSCR but aroused from findings of GWAS are summarized below.

**Table 2:** Major GWAS loci associated with HSCR.

**Gene**	**SNP**	**Population**	**Relevant information**	**Reference**
**Common variants (MAF > 0.05) with strong association**				
*RET*	rs2505998 (A/G)^a^	• Asian • European • Danish • Swedish • Finnish	• Strong effect: OR^b^ = 3.57–7.49 in different populations • Predicted to affect the expression of *RET*	(108, 111)
*NRG1*	rs7005606 (G/T)^c^	• Asian • European	• Moderate effect: OR = 2.12 (95% CI: 1.70–2.63; *p* = 1.11 × 10^−11^) in Asians, OR = 1.64 (95% CI: 1.25–2.15; *p* = 4.0 × 10^−4^) in Europeans • Predicted to affect the expression of *NRG1*	(111)
**Low-frequency variants (MAF 0.01–0.05) with strong association**				
*RET*	rs9282834 (A/G)	Asian	• Exonic missense but predicted to be benign • Strong effect: 10-fold increase of HSCR risk when the risk allele exists in compound heterozygous with the risk allele of rs2435357 • May interrupt *RET* expression or function	(111)
*RET*	rs144432435 (T/C)	• Danish • Swedish	• Strong effect: OR = 6.6 (*p* = 7.7 × 10^−10^) • Mechanism of independent effect is unknown	(108)
*SEMA3C/D*	rs80227144 (A/C)^d^	• European • Danish • Swedish • Finnish	• Moderate to strong effect: OR = 1.88 to 5.2 in different population • May modulate activity of transcription factors GATA6 and SOX7 • Monomorphic and no association in Asians	(108, 110, 111)

a *The risk allele A of rs2505998 is in LD with risk allele T of rs2435357 (r^2^ = 0.98)*.

b *OR, odds ratio*.

c *The risk allele G of rs7005606 is in LD with risk allele G of rs7835688 (r^2^ = 0.71 in East Asians and r^2^ = 1 in Europeans of the 1000 Genomes Project)*.

d *The effect allele A of this SNP is in phase with the common risk allele C of rs11766001 in the original European GWAS by (110). The GWAS array in the study by (108) did not include rs80227144. The lead SNP in the study was rs117617821 (C/T), which is in high LD with rs80227144 (r^2^ = 0.97)*.

### Neuregulin Signaling

Neuregulins (NRGs) are a family of growth factors that stimulate receptor tyrosine-protein kinase erbB (ERBB receptors) and are important regulators of neuronal migration and glia formation (112). Strong association of common SNPs (rs7835688 and rs16879552) lowering expression of *NRG1* was first identified in a GWAS on Chinese population and was widely replicated across Asian studies (102, 104, 105, 113–115). Interestingly, the association was also reported in some Caucasian studies albeit with smaller and variable effect size (108, 110, 111). Following the GWAS discovery, patient-specific rare damaging coding variants were found in both Chinese and European sporadic HSCR cases. Functional analyses of these variants showed aberrant expression and uneven intracellular distribution of the mutant NRG1 proteins, proving that not only common variants but also rare coding variants in *NRG1* can increase HSCR risk (27, 106). Besides *NRG1*, genome-wide copy number variation (CNV) analysis discovered the association of intronic deletions and duplications in *NRG3*, a paralog of *NRG1*, with HSCR on the same Chinese SNP array dataset (116). The recent population-based WGS study further identified a fourfold increase in the number of damaging rare variants in *ERBB2*, encoding the receptor for NRG1, in patients with HSCR compared to the controls (16). Altogether, these findings firmly established NRG-ERBB as one of the core HSCR pathways with significant contribution of both common and rare variants to disease pathogenesis.

### Semaphorin Signaling

Semaphorins are extracellular signaling molecules that can directly bind to several receptor protein families particularly plexins and neuropilins. Semaphorins are important regulators of axon guidance and neural crest cell migration (117). Their involvement in HSCR pathogenesis has only been brought to focus with the identification of GWAS significant association signals within the intergenic region of *SEMA3* gene cluster from the European and Danish GWASs (108, 110). Comparing the temporal and spatial localization with Ret in mouse ENS, *SEMA3D* and *SEMA3C* were considered to be the most likely candidate gene targets of the association in the cluster. Their roles in HSCR were further supported by the plausible interaction with *RET* such that co-knockdown of both *sema3c/d* and *ret* gave a more severe, aganglionic phenotype in zebrafish model. Recently, a twofold excess of rare deleterious variants in *SEMA3C* and *SEMA3D* was also observed in patients from a WES study (18).

### Hedgehog and Notch Signaling

Hedgehog (Hh) signaling is mediated by transmembrane proteins Patched (PTCH1) and Smoothened (SMO) and the downstream effector GLI transcription factors. Notch receptors (NOTCH1 and NOTCH2) are transmembrane proteins activated by their ligands (DLL1 and DLL3). They are crucial regulators of ENS development (29), and key members of these pathways were suspected to be involved in HSCR pathogenesis. Using the Chinese GWAS data (102), a significant epistatic interaction between genes of these pathways (particularly driven by *PTCH1* and *DLL3* SNPs) conferring higher risk to HSCR was discovered (29). Functional analysis validated the interaction between the two signaling pathways and the Hedgehog/Notch interaction was shown to coordinate the proliferation and differentiation of ENCCs. Another study on an independent set of Chinese patients also suggested that common variants in *PTCH1* may predispose to HSCR (118). Later, using targeted sequencing in 20 Chinese sporadic patients with HSCR devoid of *RET* coding sequence mutations, four rare coding variants in *GLI1, GLI2*, and *GLI3* were identified, and these mutations were demonstrated to enhance GLI transcriptional activity (119). Finally, as mentioned earlier, inherited pathogenic variants of *IHH* and *GLI3* genes, together with a *de novo GDNF* in-frame deletion, led to the manifestation of HSCR phenotype in one branch of a Dutch multiplex family, whereas the *RET* coding variant and intron-1 variant were responsible for the disease manifestation in another branch (28). These findings suggest that, although the reported variants in Hedgehog and Notch signaling pathway genes in patients with HSCR contribute to a small fraction of patients at present, more patients may be expected to be reported in the future especially with the increasing application of NGS and under oligogenic model harboring multiple variants in these genes.

### Epistasis (Genetic Interaction) Is an Important Part of the Genetic Architecture of HSCR

Summarizing findings of GWAS studies, it becomes increasingly apparent that epistasis is an important component of the genetic architecture of HSCR, which contributes substantially to the modified penetrance of disease causal variants. In particular, the common *RET* enhancer variant (rs2435357) plays a pivotal role in epistatic interaction within and between HSCR genes and represents the main contributor to the sensitized genetic background of patients.

Within the *RET* locus, synergistic interaction between rs2435357 and other common/low-frequency/rare variants was observed. As reported in the transethnic meta-analysis, the low-frequency, Asian-specific missense variant (rs9282834) encoding RET D489N has no effect on HSCR risk alone (OR = 1.1); however, when it occurred *in trans* with rs2435357 (OR = 3.2), D489N increased risk of HSCR by at least 10-fold (OR = 16.7/26.7 in Chinese/Korean, respectively) (111). In addition, in the Chinese S-HSCR WGS study, the genetic effects of other rare *RET* coding mutations were largely modified to different degrees depending on the predicted pathogenicity in the presence of the common risk alleles. Again, no increase in disease risk was detected for individuals carrying only a single *RET* missense damaging or benign mutation; however, for individuals heterozygous with rs2435357, damaging missense *RET* mutations conferred ~5-fold increase in risk of HSCR on top of the effect of rs2435357. Similarly, for individuals with at least two high-risk alleles occurring *in trans* (i.e., rs2435357 TT or compound heterozygous for rs2435357 T allele and rs9282834 A allele), a non-damaging *RET* missense mutation also conferred a twofold increase in disease risk additionally. On the other hand, three *cis*-acting regulatory variants in three distinct enhancers of *RET* [rs2506030 (G/A), rs7069590 (T/C), and rs2435357 (T/C)] were shown to increase HSCR risk synergistically in patients of European ancestry (109). Functional studies have provided valuable biological insights underlying the genetic interaction. These risk alleles might disrupt the binding sites of RARB, GATA2, and SOX10, respectively, and their combined effect significantly dysregulated the expression of *RET* and other functionally related genes in the gene regulatory network through positive and negative feedback. These results altogether demonstrated how the *RET* enhancer allele may modify the penetrance of other coding variants or amplify its effect in conjunction with other enhancers to affect the phenotypic expression.

Moreover, the effect of variants in other HSCR genes may also depend on the *RET* genetic background. Interestingly, both of the new GWAS significant loci were shown to interact with *RET*. A genetic interaction between *RET* and *NRG1* was observed (interaction *p* = 0.0095), in which the odds ratio increased by twofold for the *RET* rs2435357 risk genotype (TT) in the presence of *NRG1* rs7835688 heterozygote. Such genetic interplay was later confirmed functionally by showing that Nrg1 inhibited the Gdnf-induced neuronal differentiation and Gdnf negatively regulated Nrg1 signaling by downregulating the expression of its receptor, ErbB2 (120). Likewise, the frequency of *RET* rs2435357 was higher for subjects with the *SEMA3C/D* rs12707682 risk allele, which is in line with the synergistic effects on gut innervation observed by co-knockdown of *sema3c/d* and *ret* in the zebrafish model. In mice, the reduced dosage of *Ret* or *Ednrb*, the second most mutated HSCR gene, independently yielded no obvious ENS phenotype, albeit combining the two oligogenic-null heterozygote models gave rise to aganglionic phenotype (121). In summary, these findings implied that the penetrance of mutations/functional variants in other HSCR susceptibility genes may also be modulated by *RET* variants and that a sensitized background of aberrant *RET* expression might be necessary for other genetic factors to act upon for disease manifestation (122).

## Post-GWAS: Identification of HSCR-Associated Rare Variants and Genes by Unbiased Approach Using NGS Technology

The decreasing cost and hence increased adoption of NGS is expected to lead to a new era in genetic analysis of HSCR. WES and WGS studies investigating HSCR-associated rare variants in an unbiased manner are emerging and new biological insights not limited to differentiation, proliferation, and migration properties of the ENCCs begin to unveil.

### Aberrant Extracellular Matrix (ECM) Composition Involving Focal Adhesion/ECM–Receptor Interaction

During the ENS development, ENCCs migrate from the neural tube and enter the foregut, and then migrate along the gastrointestinal tract in a rostrocaudal direction. The directed cell migration requires a coordinated interaction with the microenvironment including the ECM. Theoretically, genetic defects perturbing the interaction between cell and ECM may affect the migration and colonization of the ENCCs. In a recent WGS study on the severe forms of HSCR, genes with rare *de novo*, recessive or digenic variants in patients with L-HSCR/TCA were shown to be enriched for ECM–matrix receptor interaction (19). Furthermore, a significant enrichment of damaging rare variants in genes encoding cell-adhesion proteins, *ITGB4* (Integrin beta-4) and *PTK2/FAK* (Focal adhesion kinase), was identified in an independent cohort of patients with S-HSCR (16). Integrins are a large family of cell surface receptors that connect ENCCs to the ECM. Upon activation, integrins undergo conformational change to recruit signaling molecules such as FAK and vinculin (VCL), a membrane-cytoskeletal protein in focal adhesion plaques, for the cell–matrix adhesions. Interestingly, a mutation in *VCL* (M209L) was also found in a Chinese patient with S-HSCR from integrative WES and transcriptomic analysis (32). Subsequent CRISPR/Cas9-mediated correction of the mutation in patient-specific induced pluripotent stem cells (iPSCs) efficiently rescued the differentiation and migration defects of the iPSC-derived ENCCs. In fact, abnormalities in the ECM composition in the affected bowel of patients with HSCR were noted decades ago (123). ECM proteins, such as laminins, collagens, tenascin, and fibronectin, were functionally shown to be involved in ENS development (124–126). The hypothesis that genetic defects affecting ECM composition underlie HSCR is further supported by the mouse models in which (1) loss of β1 integrin in ENCC resulted in colonic aganglionosis (127) and (2) lineage-specific upregulation of *Col6a4* in Holstein mouse model increased total collagen VI protein levels in the ECM and resulted in slower migration of ENCCs and decreased extent of bowel colonization (128).

### Neuronal Death Involving the BACE1–APP–BACE2 Pathway

Another novel finding from the NGS study was the stark excess of rare protein-altering variants in β-secretase 2 gene (*BACE2*) identified from patients with S-HSCR (16). *BACE2* is a homolog of *BACE1* encoding a protease that cleaves the amyloid precursor protein (APP) in the beta amyloid (Aβ) region and prevents its formation. Accumulation of Aβ induces neuronal death, representing the underlying cause of Alzheimer's disease. Using the iPSC platform, a patient-specific *BACE2* rare variant was demonstrated to significantly reduce the APP processing activity of BACE2 and resulted in accumulation of Aβ and thereby apoptosis of enteric neurons. Similarly, correction of the mutation using a genome editing approach ameliorated the apoptotic phenotype. Together with the marginal association of common variants of *PLD1* that may negatively regulate Aβ formation (16, 129, 130), these findings shed light on the important role of APP processing in HSCR pathogenesis.

### Other New Findings From NGS Studies

Up till now, many other genes have been reported to be associated with HSCR from trio-based and case–control NGS studies, albeit these studies are more focused on the coding region. Functional characterization of all these genes remains a daunting task—even with the use of CRISPR/Cas9-mediated knockout or morpholino-mediated knockdown in zebrafish models in a relatively fast manner, not to mention elucidating the underlying disease mechanisms. Some of these new genes with functional support for their roles in ENS development include *DENND3, NCLN, NUP98*, and *TBATA* discovered from *de novo* mutation analysis of L-HSCR trios (31) and *ACSS2, ENO3, SH3PXD2A*, and *UBR4* identified from the case–control WES study (Table 1) (18); however, how these genes predispose or cause HSCR remains to be explored. Compared to coding variants, identifying HSCR-associated functional non-coding variants from WGS studies is even more challenging and requires the development of novel analytical methods and integration of multi-omics data to facilitate their discoveries. A new framework, named MARVEL, has recently been developed and used together with functional analysis in human stem cells to identify several novel disease-associated regulatory elements, further highlighting two HSCR candidate genes—*RASGEF1A* and *PIK3C2B* (131).

## Role of CNVs and Chromosomal Anomalies

Besides rare coding and common variants, CNVs and chromosomal abnormalities have also been frequently reported in HSCR cases. Chromosomal anomalies have been reported in up to 12% of patients with HSCR, although Down syndrome (trisomy 21) alone can be found in as much as 10% of HSCR cases, which increases HSCR risk by 50- to 100-fold (132, 133). In fact, these chromosomal anomalies have facilitated the discovery of several classical HSCR genes. CNVs that are beyond the resolution of conventional cytogenetics may contribute to the missing heritability of HSCR. These groups of CNVs are becoming more and more detectable with the advent of SNP arrays and NGS as well as a plethora of bioinformatic tools and algorithms for accurately detecting CNVs (134–137). Importantly, there appears to be a higher burden of rare CNVs in patients with HSCR compared to controls and larger CNVs in syndromic HSCR compared to isolated HSCR (116, 138, 139). Besides those affecting known HSCR genes (18, 19, 116, 139, 140), several other genic CNVs have also been detected in patients with HSCR lately, whose contribution to HSCR are yet to be functionally characterized—though many of these are also associated with other neurodevelopmental disorders (18). Among these, deletions in 16p11.2 appear to be particularly interesting as at least six patients with HSCR have been reported to have deletion in this locus (18, 141). However, there appears to be no common overlapping region deleted in all the reported patients with HSCR. Deletion and duplication of 16p11.2 are also implicated in intellectual disability and psychiatric disorders (142); however, none of the genes in this region have yet been characterized in animal models with respect to their roles in ENS. It is possible that one or more genes in this locus may be critical for ENS development and therefore deletion of their coding regions can result in haploinsufficiency and thus incomplete gut colonization. Alternatively, loss or disruption of their regulatory elements can lead to dysregulation of gene expression, and, when added up with the sensitized genetic background, can lead to HSCR.

## Discussion

**Figure 1:**
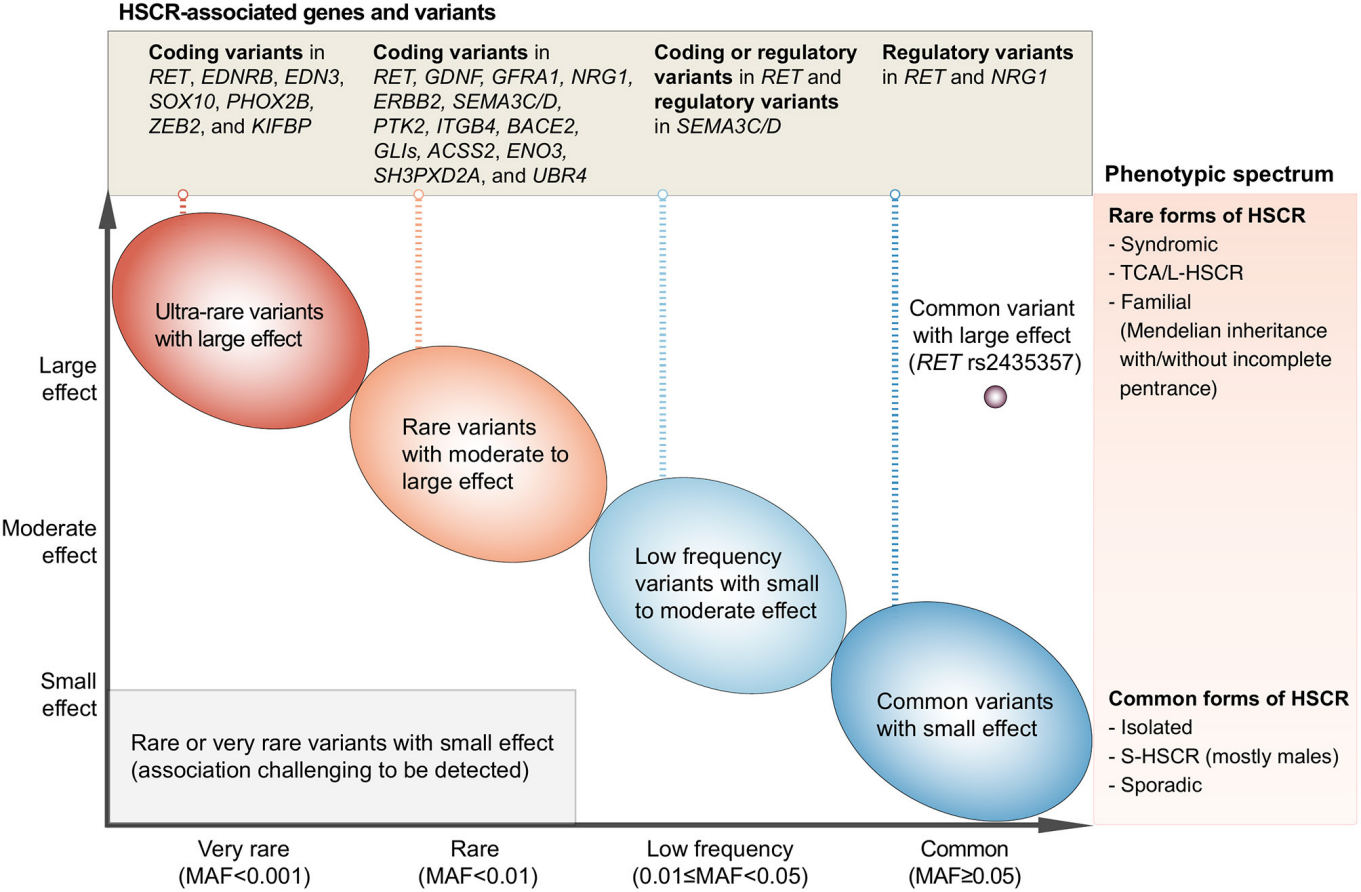
Current understanding of the genetic architecture of HSCR. The *x*-axis denotes the minor allele frequency (MAF) of variants and the *y*-axis denotes the effect size. HSCR-associated genetic variants range from variants with extremely low MAF and very large effect size (upper-left side of the plot) to the common/low-frequency variants with small to large effect size (right side of the plot). These variants have been reported in the HSCR loci shown in the upper panel and can cause HSCR with a wide spectrum of severity as denoted in the right panel. On one end of the phenotypic spectrum lies the syndromic HSCR and TCA/L-HSCR, which are the rarer and severe form of HSCR whereas on the other end lies the commonest form of HSCR, i.e., sporadic S-HSCR.

From the discoveries of HSCR-associated rare variants, common variants, and CNVs, it is evident that HSCR is a complex disease with involvement of multiple genes and pathways important for ENS development and function. Technological advances in genotyping and sequencing surmount the knowledge-intensive limitations of candidate gene approach and revolutionize genetic studies on HSCR, with disease-associated variants and genes being identified at an accelerated rate. In the past decade, the major genetic discoveries brought out by the hypothesis-free genome-wide approach have shed light into the genetic landscape and architecture of HSCR (Figure 1).

A varying degree of susceptibility to HSCR exists in the general population. This variation is largely due to the combinatorial effects of common, low-frequency, and rare inherited variants with increasing effect size (Figure 1; from right to left, from bottom to top). Indeed, the variable expressivity and phenotypic variability among carriers of the same genetic variant suggest a strong predisposition in the genetic background. The genetic findings reviewed here elegantly illustrate that common HSCR-associated variants, particularly in the major HSCR gene, *RET*, contribute not only additively but also synergistically to predisposition to HSCR. These common variants can provide a sensitized genetic background that modifies penetrance of rare disease-causing variants and, when the liability threshold is surpassed, will result in clinical manifestation. For example, a highly damaging ultra-rare loss-of-function variant in a key ENS gene (e.g., *SOX10*) is sufficient to cause the rarer form of the disease—i.e., L-HSCR/TCA—in a person with less sensitized genetic background. On the other hand, for a rare variant with moderate effect size, a highly sensitized genetic background is needed for the phenotypic expression of HSCR. Such joint and epistatic effects between and within regulatory and coding variants represent the integral component of the genetic architecture of HSCR, particularly for sporadic S-HSCR.

**Figure 2:**
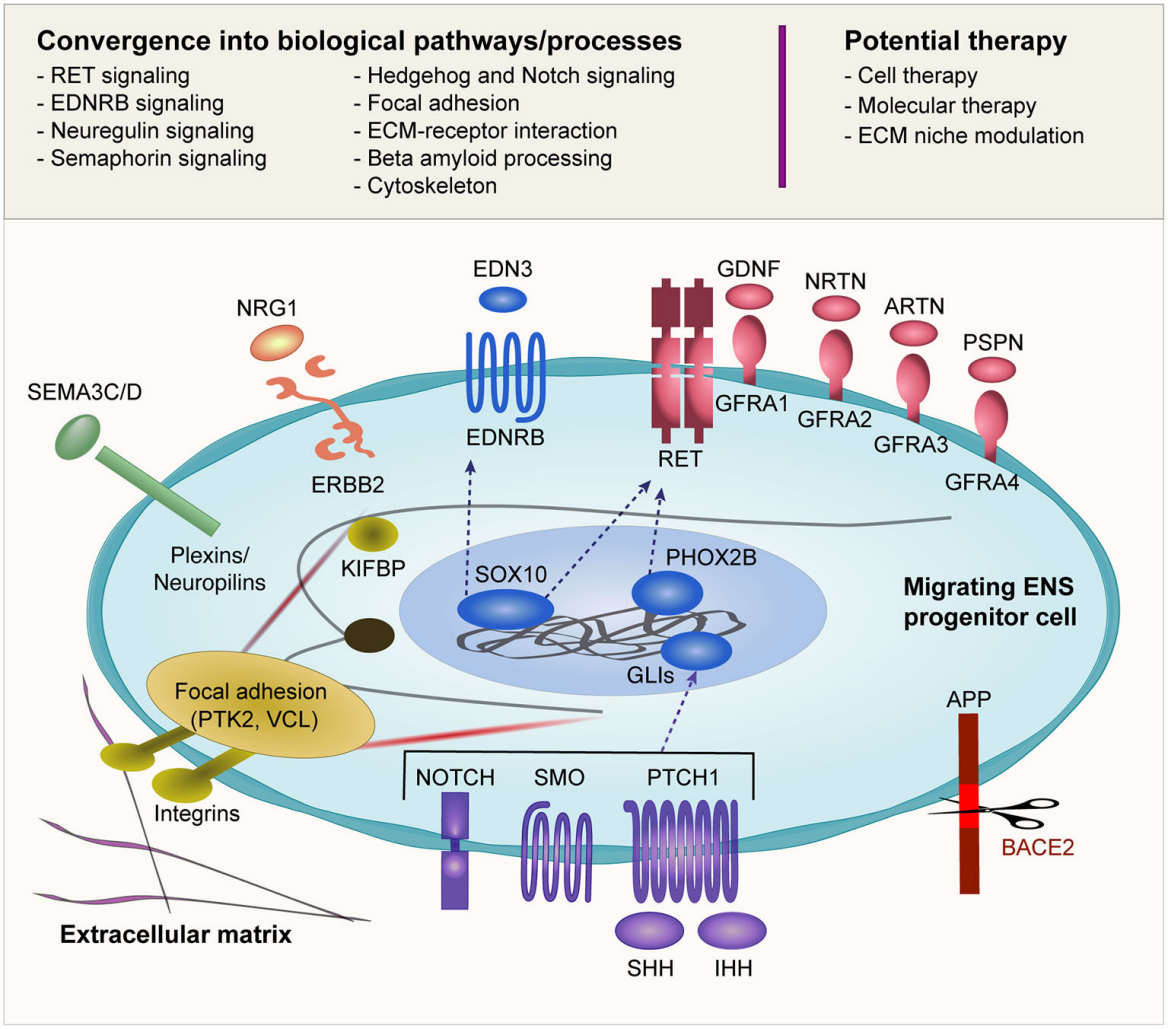
Biological pathways implicated in HSCR. Many of the reported HSCR-associated genes converge into several biological pathways as shown in this figure. The schematic diagram illustrates an ENCC migrating through the gut and its interaction with the surrounding environment including the ECM. The ENCC receives cues from the surrounding environment through different signaling molecules that regulate the cellular machinery as well as feedback interaction with the surrounding niche. The potential therapeutic approaches that are currently under investigation are also shown.

Another interesting point to note is that the biological pathways linked to HSCR are not independent of each other (Figure 2). Rather, there is considerable epistasis and crosstalk between these pathways both statistically and biologically, and a dynamic interaction of these pathways orchestrates the ENS development. A deeper understanding of the spatiotemporal crosstalk as well as the interaction between the migrating ENS precursor cells and the extracellular niche is warranted.

Despite the significant advancement in our understanding of HSCR genetics from the past three decades of research, the translation of these findings to clinical utility has yet to be established. Genetic risk prediction has potential clinical impact of assisting diagnosis, providing genetic counseling, informing treatment options, or predicting prognosis/complication/survival. Currently, there is no evidence-based consensus guideline for genetic testing of HSCR under clinical setting. The necessary first step for the clinical implementation of genetic risk prediction can be prioritized on the HSCR patient subgroups in which the phenotypic presentation is presumed to be caused by high penetrant pathogenic genetic variants and where the incremental benefits on clinical care and decision-making are optimal. Clinical genetic testing can first be considered in the case of syndromic presentation where a genetic diagnosis of a syndrome may lead to the discovery of additional anomalies in other organs and thereby enhance clinical management to improve patient care. Another potential application of clinical genetic testing can be in multiplex family where identification of segregating pathogenic variant may assist recurrence risk prediction and therefore allow genetic counseling. However, its clinical utility on non-syndromic and sporadic patients remains to be explored. Many genetic studies have demonstrated unequivocally that genetic variants of wide frequency spectrum (coding, regulatory, and CNV/chromosomal anomalies) can capture the genetic susceptibility to HSCR albeit with low to moderate discriminative power (18). Sophisticated methods combining polygenic risk of these genetic variants may leverage the performance of risk prediction, and such polygenic model may be applied for genetic testing of the major HSCR subgroup with high genetic heterogeneity. One powerful application of polygenic risk prediction is the potential ability to predict complications; however, up till now, it remains largely unknown if there exists any gene or variant significantly associated with prognosis or complication of HSCR (e.g., enterocolitis, severity of constipation, or incontinence following surgery). Further research should be encouraged to address these knowledge gaps. With the decreasing cost of NGS, WES and preferably WGS can be used in a research setting for genetic profiling. Whenever possible, parents should be included in genetic studies to facilitate the interpretation of variants. For each patient, comprehensive profiling of coding and non-coding variants, SNPs/Indels, and CNVs/chromosomal anomalies should be carried out. Together with other omics approaches (e.g., transcriptomics) and detailed phenotyping of the patients, more HSCR-associated candidate genes and biological pathways, and their differential contribution to the disease severity and complications can be discovered. In parallel, novel therapeutic options should be explored, e.g., stem cell therapy to regenerate the ENS, molecular therapy to modulate ENS formation or ECM niche manipulation, or correction of the culprit mutation with genome editing. While these alternative therapeutic approaches are still in the rudimentary stage, we envision a future where genetic testing would impact clinical care for most of the patients with HSCR by assisting diagnosis and clinical care, predicting complications, and, more importantly, providing alternative treatment options with much better clinical outcome.

## Author Contributions

AK and CT wrote the manuscript. PT did the final critical review of the manuscript. All authors contributed to the article and approved the submitted version.

## Conflict of Interest

The authors declare that the research was conducted in the absence of any commercial or financial relationships that could be construed as a potential conflict of interest.

## Publisher's Note

All claims expressed in this article are solely those of the authors and do not necessarily represent those of their affiliated organizations, or those of the publisher, the editors and the reviewers. Any product that may be evaluated in this article, or claim that may be made by its manufacturer, is not guaranteed or endorsed by the publisher.
